# Cardiac involvement in patients with primary biliary cholangitis: A 14-year longitudinal survey-based study

**DOI:** 10.1371/journal.pone.0194397

**Published:** 2018-03-15

**Authors:** Sainan Bian, Hua Chen, Li Wang, Yunyun Fei, Yunjiao Yang, Linyi Peng, Yongzhe Li, Fengchun Zhang

**Affiliations:** 1 Department of Rheumatology and Clinical Immunology, Peking Union Medical College Hospital, Chinese Academy of Medical Sciences & Peking Union Medical College, Beijing, China; 2 Key Laboratory of Rheumatology & Clinical Immunology, Ministry of Education, Beijing, China; Texas A&M University, UNITED STATES

## Abstract

Patients with primary biliary cholangitis (PBC) can have extrahepatic manifestations. However, data about cardiac involvement of PBC is limited. We aimed in this study to analyze the clinical characteristics in patients with PBC complicated with and without cardiac involvement, and the risk factors of cardiac involvement in PBC. PBC patients admitted to Peking Union Medical College Hospital between January 2002 and February 2016 were consecutively enrolled. Structured interview, systemic rheumatologic examination, and laboratory tests were conducted for each patient, and risk factors of cardiac involvement were analyzed by comparing patients with and without cardiac involvement. In total, 580 PBC patients were enrolled, and cardiac involvement was identified in 24 patients (4%), with 11 male (46%) and a mean age of 57±8 year. Cardiomyopathy and arrhythmias were presented in 17 (70.8%) and 21 (87.5%) patients, respectively. Patients with cardiac involvement were more frequently male (46% vs. 11%, P<0.01), complicated with inflammatory myopathy (IM) (58% vs. 1%, P<0.01), and had a longer disease course (median, 72 vs 24 month, P<0.01). Furthermore, concomitant IM was the independent risk factor of cardiac involvement in PBC (OR = 77.333, 95% CI: 23.704–252.294). Cardiac involvement was a rare complication of PBC, which was more frequently observed in male or long-course patients. Importantly, concomitant IM was the strong independent risk factor of cardiac involvement in PBC. Given cardiac involvement is a serious complication, thorough evaluation of cardiac manifestation in high-risk PBC patients is highly recommended.

## Introduction

Primary biliary cholangitis (PBC), which was also previously known as primary biliary cirrhosis (PBC), is an autoimmune disease characterized with elevated alkaline phosphatase, positive anti-mitochondrial antibody (AMA), and destruction of small intrahepatic bile ducts in histology. PBC predominantly affects women, and are usually presented in the fifth or sixth decade of life[1].

PBC primarily involves the liver, but extrahepatic manifestations are increasingly reported. Among them, interstitial lung disease and pulmonary hypertension are the most prevalent manifestations, with an estimated prevalence of 15.7% and 11.8%, respectively[2, 3]. Cases of nephropathy are also reported[4, 5]. Cardiac involvement, including cardiomyopathy and arrhythmias, such as ventricular tachycardia, and conduction block, is increasingly reported in PBC patients recently[6–10].

Although Cardiac involvement is a critical even fatal complication, limited data are currently available regarding the incidence and clinical features of cardiac involvement in PBC patients. We have observed several PBC cases of severe heart failure in clinical practice, which suggested a possible link between PBC and cardiomyopathy. To address this issue, we employed a large cohort of consecutive PBC patients and analyzed the clinical characteristics of PBC patients with and without cardiac involvement, to identify potential risk factors associated with cardiac involvement. In addition, we reviewed and summarized the cases reported of cardiac involvement in PBC.

## Methods

### Patients

PBC patients who were admitted to Peking Union Medical College Hospital (PUMCH), Beijing, China between January 2002 and February 2016 were enrolled consecutively.

The diagnosis of PBC was made depended on the criteria proposed by American Association for the Study of Liver Diseases: chronic cholestasis with increased serum alkaline phosphatase level and/or γ-glutamyl transpeptidase level; positive anti-mitochondrial antibodies (AMA); histological features in the liver that are indicative of the diagnosis[11, 12]. Other obstructive jaundice diseases were excluded.

A structured interview, systemic rheumatologic examination, and laboratory tests (including liver and renal function, autoantibodies, myocardial enzyme, electrocardiogram, and echocardiography) were performed for each. Clinical data including demographics, clinical manifestation, laboratory evaluation, and liver and myocardium histology (if available) were collected.

Cardiac involvement was defined as presence of cardiomyopathy (left ventricular or biventricular systolic dysfunction)[6, 13, 14]and arrhythmias (mainly ventricular arrhythmia, or conduction defects). Arrhythmias were diagnosed by two cardiologists according to the manifestations of electrocardiogram (ECG) and Holter monitor[15]. In patients with cardiac involvement, contrasted-enhanced coronary artery tomography or angiography were performed to evaluate coronary artery disease (CAD).

In addition, polymyositis (PM) and dermatomyositis (DM) were diagnosed according to the Bohan and Peter criteria[16].

The study was reviewed and approved by the Institutional Review Board of Peking Union Medical College Hospital, and waiver of consent was achieved since the data were analyzed anonymously.

### Statistical analysis

Kolmogorov-Smirnov test was used to check if the variables followed normal distribution. Measurement data of normal distribution were expressed by mean±standard deviation (SD), and measurement data of non-normal distribution were expressed by median and interquartile range (IQR). Enumeration data were expressed by percentage. Continuous and categorical variables were compared using the independent samples t test and Pearson χ^2^ test, respectively. Potential factors associated with cardiac involvement were further evaluated with stepwise multivariable logistic regression models. A 2-sided p value <0.05 was considered statistically significant. Statistical analysis was performed by SPSS 16.0 (SPSS Inc, Chicago, IL, USA).

## Results

### Baseline demographic data of PBC patients

In total, 580 PBC patients were enrolled, with a mean age of 56±12 year and 88% of female. AMA was positive in 530 patients (91%), and presence of M2 subtype of anti-mitochondrial antibody was observed in 528 patients (91%). Hepatic histology was available in 140 patients (Table 1). Polymyositis (PM) were diagnosed in 14 patients and muscle biopsy was performed in 11 patients.

**Table 1:** Demographics, clinical manifestations, and laboratory examinations of PBC patients with or without cardiac involvement.

Group	Cardiac involvement (n = 24, 4%)	No cardiac involvement (n = 556, 96%)	P value
Age (yr)	57±8	56±12	0.621
Female-n (%)	13 (54)	495 (89)	<0.01
Concomitant IM-n (%)	14 (58)	7 (1)	<0.01
Disease duration (month)-median (IQR)	72 (24–120)	24 (6.7–60)	<0.01
Autoantibodies			
AMA-n (%)	22 (92)	508 (91)	0.959
AMA-M2-n (%)	23 (96)	505 (91)	0.401
ACA-n (%)	3 (13)	135 (24)	0.185
Liver function tests			
Albumin (g/L)	39±5	36±6	0.060
PT (second, s)	12.2±2.8	12.0±1.6	0.534
ALP (U/L)	178 (153–410)	237 (151–423)	0.388
GGT (U/L)	193 (111–426)	200 (104–424)	0.939
TBil (μmol/L)	16.2 (10.7–24.6)	18.7 (10.8–32.8)	0.307
Cirrhosis-n (%)	5 (20.9)	162 (29.1)	0.379
Hepatic histology-n (%)	2 (8.3)	143 (25.7)	
PBC I-II	2/2 (100)	115/143 (80.4)	0.486
PBC III-IV	0 /2(0)	28 /143 (19.6)	0.486
IgM (g/L)	3.03 (1.45–4.78)	3.27 (1.88–4.77)	0.708
IgG (g/L)	17.13±6.33	17.41±7.00	0.856
IgA (g/L)	2.47 (1.87–3.32)	3.09 (2.24–4.37)	0.039
ESR (mm/h)	50±27	47±31	0.618
CRP (mg/L)	4.33 (2.15–9.97)	3.43 (1.47–9.53)	0.429
Hyperlipemia	4 (16.7)	149 (26.8)	0.270
T2DM	2 (8.3)	77 (13.8)	0.441
Hypertension	9 (37.5)	313 (56.3)	0.070
Overweight or obese	4 (16.7)	63/348 (18.1)	0.859

IM: Inflammatory myopathy; AMA: Anti-mitochondrial antibody; AMA-M2: M2 subtype of anti-mitochondrial antibody; ACA: Anti-centromere antibody; PT: Prothrombin time; ALP: Alkaline phosphatase; GGT: γ-glutamyl transpeptidase; TBil: Total bilirubin; IgM: Immunoglobulin M; IgG: Immunoglobulin G; IgA: Immunoglobulin A; ESR: Erythrocyte sedimentation rate, normal range <20mm/h; CRP: C-reactive protein, normal range<3mg/L; T2DM: Type 2 diabetes mellitus.

### Cardiac involvement in PBC

Cardiac involvements were documented in 24 patients (4.1%), including 17 patients (70.8%) with cardiomyopathy, 21 patients (87.5) with arrhythmias, and 14patients (58.3%) with both cardiomyopathy and arrhythmias. One patient with CAD was excluded from the study.

Cardiac manifestations were presented simultaneously with PBC in 13 (54%) patients, and presented after PBC in 4 patients (17%) (2 to 6 years). In 7 patients (29%), cardiac manifestations were presented prior to the diagnosis of PBC. Myocardial biopsy of one patient revealed mild degeneration of myocardial tissue and infiltration of lymphocytes.

### Echocardiography

**Fig 1:**
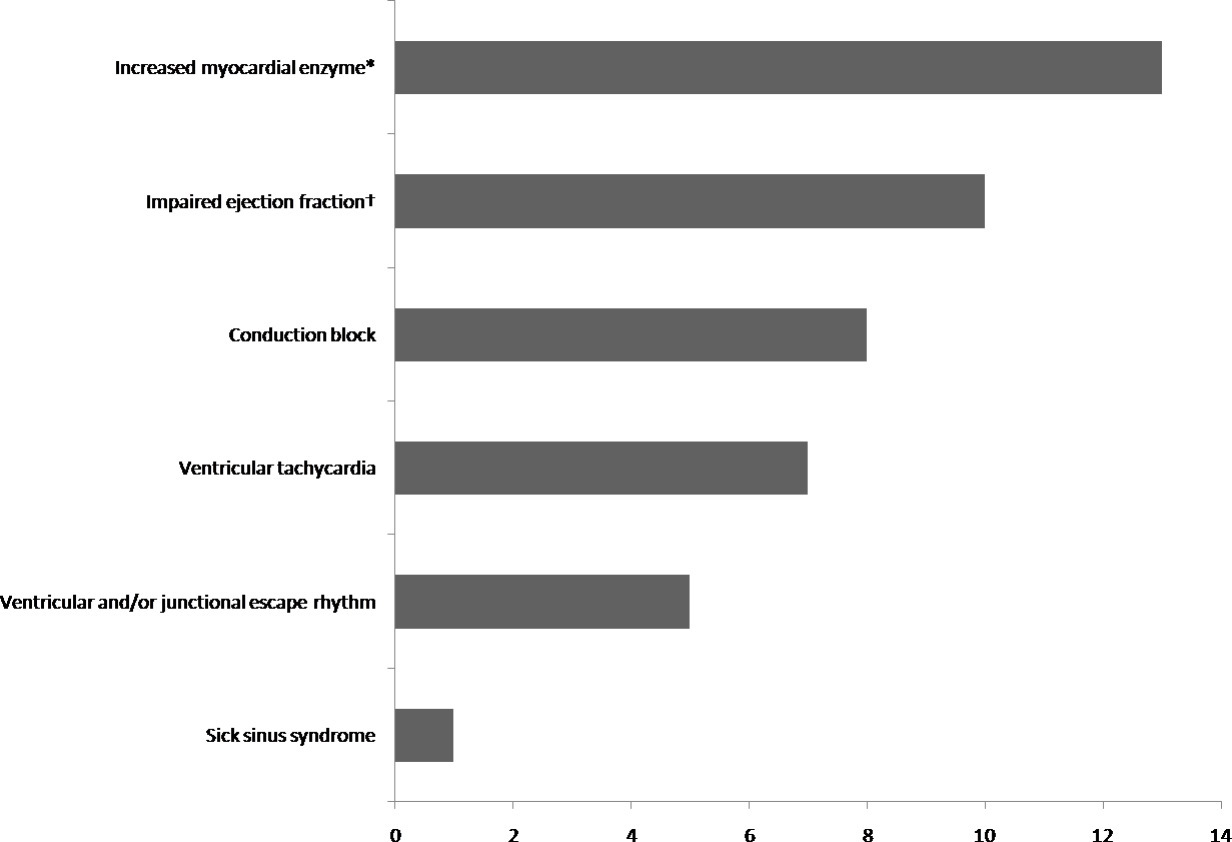
Cardiac dysfunctions in PBC patients. *Performed in 19 patients, defined as CK>198U/L, CKMB>6.3ug/L or cTnI>0.04ug/L. †defined as EF<50%.

Impaired ejection fraction (EF) (<50%) was observed in 10 patients (41.7%), with a mean EF of 39±15%.Whole heart enlargement was observed in 9 patients (37.5%) (Fig 1).

### Myocardial enzyme of PBC patients with cardiac involvement

Among the 24 PBC patients complicated with cardiac involvement, increased myocardial enzyme were observed in13 out of 19 patients (Fig 1).The median creatine kinase (CK) level was 887 U/L (IQR 384–1181) (normal range 18–198 U/L). The median creatine kinase MB (CKMB) level was 23.8μg/L (IQR 8.9–41.1, range 7.0–199.0) (normal range 0.6–6.3). Median cardiac troponin I (cTnI) was 0.15 (IQR 0.11–0.19, range 0.06–16.73)μg/L (normal range 0–0.04).

### Electrocardiogram (ECG) and Holter monitor

Among the 21 patients with arrhythmias, conduction block (n = 8, 38.1%), ventricular tachycardia (n = 7, 33.3%), ventricular and/or junctional escape rhythm (n = 5, 23.8%), were the most common arrhythmias, followed by sick sinus syndrome in 1 patient (4.8%). Eight patients were complicated with atrial fibrillation and atrial flutter at the same time (Fig 1).

### Comparison of cardiac involvement with non-cardiac involvement in PBC

Patients with cardiac involvement showed male predominance (46% vs11%, P<0.01), higher incidence of inflammatory myositis (58% vs 1%, P<0.01), longer disease course (72 (24–120) vs24 (6.7–60) months, P<0.01) and lower level of Immunoglobulin A (IgA) (2.47 (IQR 1.87–3.32) vs 3.09 g/L (IQR 2.24–4.37), P = 0.039).

Age, autoantibodies profile and liver function tests were comparable between two groups. A modest trend of higher erythrocyte sedimentation rate (ESR) and C-reactive protein (CRP) in patients with cardiac involvement were noted, but the differences were not statistically significant. Cirrhosis was documented in 5 patients (20.9%) with cardiac involvement and 162 patients (29.1%) without cardiac involvement, respectively. Hepatic histology was available in 2 patients with cardiac involvement and 143 patients without cardiac involvement. Among them, 2 (100%) patients with cardiac involvement and 115 (80.4%) patients without cardiac involvement were at stage of PBC I-II, and 28 patients (19.6%) patients without cardiac involvement were at stage of PBC III-IV (Table 1). No differences on dyslipidemia, type 2 diabetes mellitus, hypertension, and overweight or obesity were observed between two groups (Table 1).

### Risk factors of cardiac involvement in PBC Patients

We further analyzed the potential risk factors associated with cardiac involvement in PBC, including gender, concomitant IM, disease course and IgA. However, multivariate logistic regression analysis showed that concomitant IM was the only independent risk factor related to cardiac involvement in PBC patients (P = 0.000, odds ratio (OR) = 77.333, 95% confidence interval (CI): 23.704–252.294) (Table 2).

**Table 2:** Risk factors of cardiac involvement in PBC patients.

Variables	P value	(OR)	95% CI
Concomitant IM	0.000	77.333	23.704–252.294

OR: Odds ratio; CI: Confidence interval; IM: Inflammatory myopathy.

### Outcomes of PBC patients with cardiac involvement

All patients received ursodesoxycholic acid (UDCA). In addition to UCDA treatment, 20 patients with cardiac involvement received high-dose glucocorticoids, including methylprednisolone pulse therapy in 3 patients. Two patients received immunosuppressive agents. 2 patients received pacemakers and 1 patient received radiofrequency ablation. On discharge, the cardiac dysfunction was improved in 19 patients. Eight patients were lost to follow-up, and cardiac condition remained stable for the 16 patients who were followed for a median of 27.0 months (IQR 11.3–49.5). No death was occurred during follow-up period. We are continuously monitoring these patients for long-term outcome.

### Literature review

We also summarized cases reported on Pubmed[6–10]. In total, we found 11 cases reports (Table 3). The mean age was 52±9 year old, and 9 patients (82%) were female. The median disease duration was 5 years (IQR 2–10). Cardiac involvement were developed after PBC in 5 patients (45%)(range 4–10 years), and simultaneously with PBC in 5 patients (45%). All patients presented with arrhythmia, and cardiomyopathy, cardiomegaly, left ventricular hypomotility were presented in 6, 1 and 1 patients, respectively. Strikingly, all patients were complicated with myopathies, including 9 of IM and 2 of myopathy. Myocardium histology was available in 4 patients, which showed interstitial fibrosis in three cases and myositis in one case.

**Table 3:** Literature review of cases of PBC with cardiac involvement.

References	Publishing date	Country	Age(year)/Gender	Disease duration(year)	AMA	Temporal relationship of PBC diagnosis to onset of cardiac involvement^1^	Cardiac manifestations	Myocardium histology	Concomitant disease
[7]	1992	Japan	42/M	5	(+)	Simultaneously	AF, SSS, AVB	NA	PM
[8]	1993	USA	58/F	10	(+)	Simultaneously	AF, conduction disturbance, congestive cardiomyopathy	Interstitial fibrosis without inflammation	Myopathy
[8]	1993	USA	44/F	13	(+)	-120 months	Af, cardiomegaly	Interstitial fibrosis(autopsy)	Myopathy
[9]	2007	Japan	37/F	5	(+)	+5 months	AVPC, left ventricular hypomotility	NA	Chronic myositis
[9]	2007	Japan	60/F	16	(-)^2^	-36 months	MVPC, AVB, dilated cardiomyopathy	Myositis of heart muscle(autopsy)	Chronic myositis
[6]	2012	Japan	54/F	1	(+)	-84 months	Af, cardiomyopathy	NA	IM
[6]	2012	Japan	49/F	2	(+)	Simutaneously	AF, PSVT	NA	IM
[6]	2012	Japan	48/F	2	(+)	Simutaneously	AVB, cardiomyopathy	NA	IM
[6]	2012	Japan	54/F	2	(+)	-84 months	Non-sustained VT, cardiomyopathy	NA	IM
[6]	2012	Japan	59/M	5	(+)	Simutaneously	Af, cardiomyopathy	NA	IM
[10]	2012	Japan	65/F	5	(+)	-16 months	First degree of AVB	Interstitial fibrosis with infiltration of CD3-positive T cells	PM

^1^—PBC diagnosed before onset of cardiac involvement, + PBC diagnosed after onset of cardiac involvement

2: Biopsy-proven PBC.

AMA: Anti-mitochondrial antibody; M: Male; AF: Atrial fibrillation; SSS: Sick sinus syndrome; AVB: Atrioventricular block; NA: Not available; PM: Polymyositis; F: Female; Af: Atrial flutter; AVPC: Atrial and ventricular premature contractions; MVPC: Multifocal ventricular premature contraction; PSVT: Paroxysmal supraventricular tachycardia; VT: Ventricular tachycardia.

## Discussion

In this study, we investigated a large PBC cohort to characterize the clinical features and risk factors of cardiac involvement in patients with PBC. We showed cardiac involvement was a rare complication of PBC, which presented in 4% of PBC patients. PBC patients with cardiac involvement were predominantly males, with longer disease course, and frequently complicated with IM.

PBC is dominated in female (92%)[1], however, in our study, 46% of patients with cardiac involvement were male, which is much higher than PBC patients without cardiac involvement (11%). Although male gender is implicated as a risk factor of cardiac involvement in PBC, it was not confirmed in multivariate analysis. In contrast, most patients (82%) were female in the literature review. Nevertheless, our data suggested evaluation of cardiac involvement in male patients of PBC is necessary, especially in high-risk patients. Similarly, Jones DE’s study suggest left ventricular ejection time was impaired in PBC compared with healthy controls[17]. Newton JL’s study also found that symptoms of cardiovascular autonomic dysfunction were significantly more frequently reported and significantly more severe in PBC patients than in matched normal controls[18, 19]. However, hepatic function was comparable in the groups of patients in our study. Patients with cardiac involvement have much longer course (72 (24–120) vs 24 (6.7–60) month, P<0.01). Similarly, Maeda, M.H. et al also found that chronic disease courses were more frequently observed in AMA-positive IM patients than in AMA-negative IM patients[6]. Although it was not confirmed as a risk factor in multivariate analysis, for long-course PBC with suspected cardiac manifestations, a systemic cardiac evaluation is still warranted.

In our study, PBC patients with cardiac involvement presented more frequently with IM. And multivariate analysis also showed that it was a strong independent risk factor of cardiac involvement (P<0.01, OR 77.333, 95%CI 23.704–252.294). Literature review of the 11 cases were also all complicated with myopathies. Interestingly, in a212-case cohort of IM, 7patients were complicated with PBC, and cardiac involvement was found in five patients (71.4%). While among the 205 IM patients without PBC, only three (1.46%) had cardiac involvement (P<0.01)[6],which implicated that patients with concomitant PBC and IM were at high risk of developing cardiac involvement. The authors suggest that AMA is not only the markers of PBC but also the factors involved in pathogenic mechanisms. Further study is needed to elucidate the role of AMAs in the pathogenesis of cardiac involvement.

The mechanism of cardiac involvement in PBC remains elusive. In addition to periductal infiltration of lymphocytes, mainly CD4 and CD8 T cells[6], lymphocytes infiltration was also observed in skeletal muscleas well as myocardial tissue in one patient’s myocardial biopsy. And Varga et al. proposed that cardiomyopathy might be one of the types of muscle involvement in PBC[8]. Granulomatous inflammation with bile duct injury is a characteristic liver histopathological feature of PBC, which is also observed in other organs including skin[20] and lung[21]. Interestingly, Maeda, M.H. et al show granulomatous inflammation in muscle histopathology[6], which implicate that granulomatous inflammation might present in myocardial tissues of PBC. Further studies of myocardial histology might clarify this point. Moreover, autoantibodies of PBC play a potential role in the pathogenesis. The anti-mitochondrial antibody potentially disturbs cardiac energy metabolism by inhibition of nucleotide transport, and induce cytotoxic effect by cross-reacting with a Ca^2+^ channel protein[10].

Our study has several limitations. First, given myocardium biopsy is an invasive procedure, it was performed in only one patient and the cardiac histological data was largely incomplete, which might underestimate the prevalence of cardiomyopathy. Second, this study is a single-center study conducted in a national referral center of complex diseases, and consequently the cohort might not be representative of patients in general centers. Third, as this is a retrospective study, long-term prognosis and survival was unavailable.

## Conclusions

We reported a large cohort of PBC patients, and observed cardiac involvement in about 4% of patients. Patients with cardiac involvement were more frequently male, complicated with inflammatory myopathy, and had a longer disease course. Furthermore, concomitant IM was the strong independent risk factor of cardiac involvement in PBC. Given cardiac involvement is a serious complication, thoroughly evaluation of cardiac manifestation in high-risk PBC patients is highly recommended.
